# A Novel Transfer Learning Approach to Enhance Deep Neural Network Classification of Brain Functional Connectomes

**DOI:** 10.3389/fnins.2018.00491

**Published:** 2018-07-24

**Authors:** Hailong Li, Nehal A. Parikh, Lili He

**Affiliations:** ^1^Perinatal Institute, Cincinnati Children's Hospital Medical Center, Cincinnati, OH, United States; ^2^Department of Pediatrics, University of Cincinnati College of Medicine, Cincinnati, OH, United States

**Keywords:** deep learning, transfer learning, functional connectomes, resting-state functional MRI, neural networks, stacked sparse autoencoder, autism spectrum disorder

## Abstract

Early diagnosis remains a significant challenge for many neurological disorders, especially for rare disorders where studying large cohorts is not possible. A novel solution that investigators have undertaken is combining advanced machine learning algorithms with resting-state functional Magnetic Resonance Imaging to unveil hidden pathological brain connectome patterns to uncover diagnostic and prognostic biomarkers. Recently, state-of-the-art deep learning techniques are outperforming traditional machine learning methods and are hailed as a milestone for artificial intelligence. However, whole brain classification that combines brain connectome with deep learning has been hindered by insufficient training samples. Inspired by the transfer learning strategy employed in computer vision, we exploited previously collected resting-state functional MRI data for healthy subjects from existing databases and transferred this knowledge for new disease classification tasks. We developed a deep transfer learning neural network (DTL-NN) framework for enhancing the classification of whole brain functional connectivity patterns. Briefly, we trained a stacked sparse autoencoder (SSAE) prototype to learn healthy functional connectivity patterns in an offline learning environment. Then, the SSAE prototype was transferred to a DTL-NN model for a new classification task. To test the validity of our framework, we collected resting-state functional MRI data from the Autism Brain Imaging Data Exchange (ABIDE) repository. Using autism spectrum disorder (ASD) classification as a target task, we compared the performance of our DTL-NN approach with a traditional deep neural network and support vector machine models across four ABIDE data sites that enrolled at least 60 subjects. As compared to traditional models, our DTL-NN approach achieved an improved performance in accuracy, sensitivity, specificity and area under receiver operating characteristic curve. These findings suggest that DTL-NN approaches could enhance disease classification for neurological conditions, where accumulating large neuroimaging datasets has been challenging.

## Introduction

Early diagnosis and prognosis remains a significant challenge for many neurological disorders, especially for rare disorders where studying large cohorts is not possible. Integration of resting-state functional magnetic resonance imaging (rs-fMRI) techniques and machine learning algorithms is showing great promise in unveiling hidden pathological functional connectome (FC) patterns to assist early diagnosis and prediction of brain disorders (Cox and Savoy, 2003; Mourão-Miranda et al., 2005; Fan et al., 2007; Pereira et al., 2009; Anderson et al., 2011; Zhang et al., 2012; Uddin et al., 2013; Plitt et al., 2015). However, brain FC patterns analysis remains challenging due to the inherent high dimensionality of data and insufficient sample sizes (Kim et al., 2016; Suk et al., 2017).

To reduce the high dimensionality of FC patterns, a number of methods have been proposed, ranging from graph theory-based features (Bullmore and Sporns, 2009; Rubinov and Sporns, 2010) to recent deep learning approaches (Kuang et al., 2014; Kim et al., 2016; Guo et al., 2017). Several investigators have reported their attempts in applying state-of-the-art deep learning algorithms, such as deep neural network (DNN) (Hinton and Salakhutdinov, 2006; Hinton et al., 2006), on rs-fMRI data to extract high-level FC features for the classification of neuropsychiatric and neurodevelopmental disorders (Kuang et al., 2014; Kim et al., 2016; Guo et al., 2017; Heinsfeld et al., 2018). Kim et al. (2016) developed a DNN model for the classification of schizophrenia from healthy controls using FC patterns derived from rs-fMRI data; the DNN model was trained based on FC patterns from 50 schizophrenia and 50 healthy controls. Their proposed approach achieved an error rate substantially lower than support vector machine (SVM). In another study (Kuang et al., 2014), a deep belief network model, a class of DNN, was tested on three attention deficit hyperactivity disorder datasets, which contained 83, 85, and 222 subjects, respectively. The ADHD discrimination accuracy was improved compared to previous results from the ADHD-200 competition (The ADHD-200 Consortium, 2012). Guo et al. (2017) developed a DNN model with a novel feature selection method for ASD diagnosis. They applied a total of 110 sets of FC patterns extracted from the ABIDE database (Di Martino et al., 2014) to train their deep learning model. Remarkably, the deep model outperformed classical models with improvement in accuracy of 9.1%. More recently, Heinsfeld et al. (2018) reported their state-of-art results (70% on accuracy) on ASD identification by using a DNN model that was trained with the ~1,000 ABIDE subjects. Benefiting from the dimensionality reduction of DNN techniques, these studies have enhanced the performance of brain classification tasks. However, in order to take full advantage of deep learning models, a large number of samples are usually required for training the model. Unfortunately, most FC studies collect data from small samples.

While increasing a study's sample is the preferred solution, in reality it is very challenging to collect large scale neuroimaging datasets, especially ones with outcome data, because the: (1) cost of MRI scanning is high; (2) it is challenging to accumulate a large dataset for rare diseases; (3) acquiring outcome data typically requires clinical diagnoses which may require long-term follow-up of subjects. Therefore, despite a few promising reports (Kuang et al., 2014; Kim et al., 2016; Guo et al., 2017; Heinsfeld et al., 2018), the use of DNN techniques to learn brain networks for outcome prediction is still immature and have not been broadly adopted by the neuroimaging community. At present, most deep learning approaches in previous studies on FC features were developed using only modest sample sizes (Di Martino et al., 2014; Kuang et al., 2014; Kim et al., 2016; Guo et al., 2017; Heinsfeld et al., 2018). The reproducibility and generalizability of these deep learning approaches are debatable, especially since the sample size of other neurological studies are small or modest at best. In order to translate the advantages of deep learning to explicate neuronal underpinnings, there is a critical need to develop a machine learning approach with robust training methodology that is geared toward the needs of studies with limited sample sizes.

Intriguingly, much of human learning involves only a few new examples superimposed on extensive prior knowledge (Fei-Fei et al., 2006). For instance, medical students can rapidly learn certain abnormalities on brain MRI images with only a handful of examples because they are already familiar with healthy brain structures. In contrast, this may be very difficult for a non-medical student lacking previous neuroscience knowledge. Motivated by knowledge of how humans learn, several learning frameworks, such as transfer learning (Pan and Yang, 2010), one-shot learning (Fei-Fei et al., 2006), and self-taught learning (Raina et al., 2007), have been proposed. These studies focus on storing prior knowledge gained from solving previous problems and applying it to a related or totally new problem. For example, knowledge gained from learning to recognize handwritten digits (“0”–“9”) can be transferred to recognize handwritten English characters (“a”–“z”) (Raina et al., 2007). In a study from the Alzheimer's Disease Neuroimaging Initiative database, knowledge learned from 10,000 regular images was utilized to learn a sparse representation of structural brain MRI data to facilitate enhanced classification of Alzheimer's disease (AD) and mild cognitive impairment from healthy control subjects (Gupta et al., 2013). Furthermore, Cheng et al. (2018) proposed a robust multi-label transfer feature learning for early diagnosis of AD and effectively improved the performance of AD diagnosis, compared with several state-of-art methods. Recently, we successfully applied a transfer learning strategy on a stacked sparse autoencoder (SSAE) model to perform high-level feature extraction of FC patterns for early prediction of cognitive deficits in a small cohort of very preterm infants (He et al., 2018).

We hypothesized that healthy FC patterns learned from an existing large scale database could be transferred to enhance a new disease classification task that also replies on FC patterns. We propose a deep transfer learning neural network (DTL-NN) model by utilizing relatively easy-to-obtain FC patterns from a database of healthy subjects. We first employed a large database of healthy FC patterns to train a SSAE prototype, and then transferred this SSAE prototype to build a DTL-NN for other new classification tasks that had limited training samples. Different from existing deep learning approaches (Di Martino et al., 2014; Kuang et al., 2014; Kim et al., 2016; Guo et al., 2017; Heinsfeld et al., 2018), our proposed approach is based on transfer learning which exploits readily available data from existing imaging databases to pre-train a deep learning model prototype so as to aid the training process of the final model. This transfer learning strategy is able to enhance DNN classification, especially when the target dataset has a limited sample size. To test the validity of our DTL-NN framework, an international collaborative project, the Autism Brain Imaging Data Exchange (ABIDE) (Nielsen et al., 2013) was chosen as a testbed. Accurate classification of autism spectrum disorder (ASD) was our target task in this work. Specifically, we selected pre-processed rs-fMRI data from four ABIDE sites with a minimum of 60 sample sizes. The proposed DTL-NN model was trained and tested within those sites, individually. Our objective was to investigate if DTL-NN could significantly enhance ASD classification, when working with limited data as, as compared with traditional DNN (Bengio and LeCun, 2007) and support vector machine (SVM).

## Materials and methods

### Deep transfer learning neural network overview

**Figure 1:**
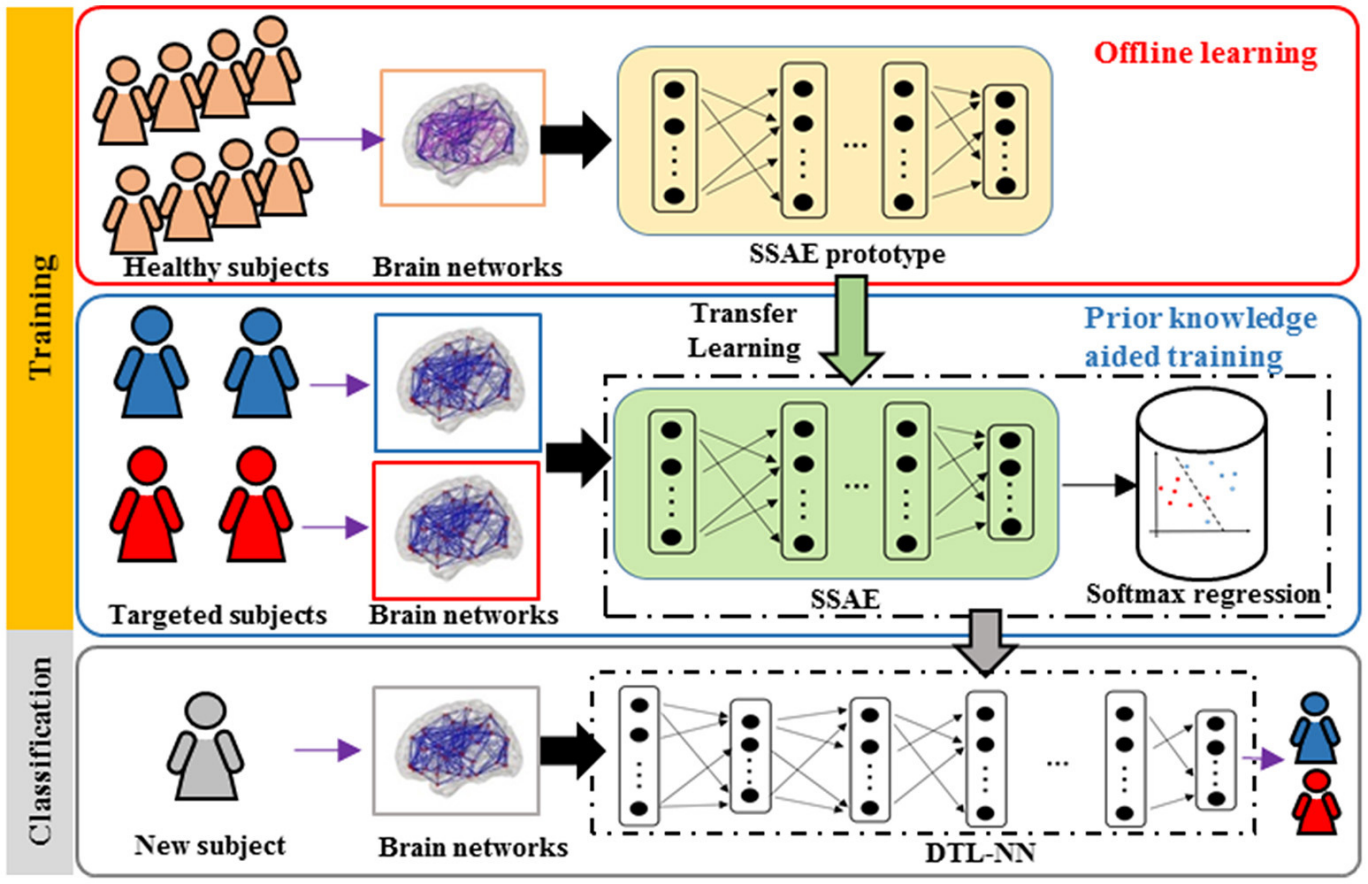
Overview of the DTL-NN framework. Training Phase: (1) A SSAE is trained in an unsupervised manner to learn healthy data in the offline learning phase (red box). (2) The learned knowledge within the SSAE is then transferred to initialize the SSAE of the DTL-NN, followed by supervised training and fine-tuning steps in the prior knowledge aided classification (blue box). Classification phase: the classification of a new subject using well-trained DTL-NN (Gray box).

An overview for the training and classification phases of our approach is summarized in Figure 1. The training phase of DTL-NN framework consists of two steps: (1) offline learning, (2) prior knowledge aided training. In step (1), we train a SSAE prototype in an unsupervised manner to learn FC patterns from a group of pre-collected healthy subjects. (Figure 1. top red panel) In step (2), the SSAE prototype could be transferred to a new independent task with limited target subjects. The SSAE prototype and a softmax regression were stacked into a DTL-NN, which is subsequently trained, aided by prior knowledge from offline learning. (Figure 1. middle blue panel) After the training phase, the well-trained DTL-NN model could be applied to classify an unknown subject into a known group (Figure 1. bottom gray panel).

### Offline learning

**Figure 2:**
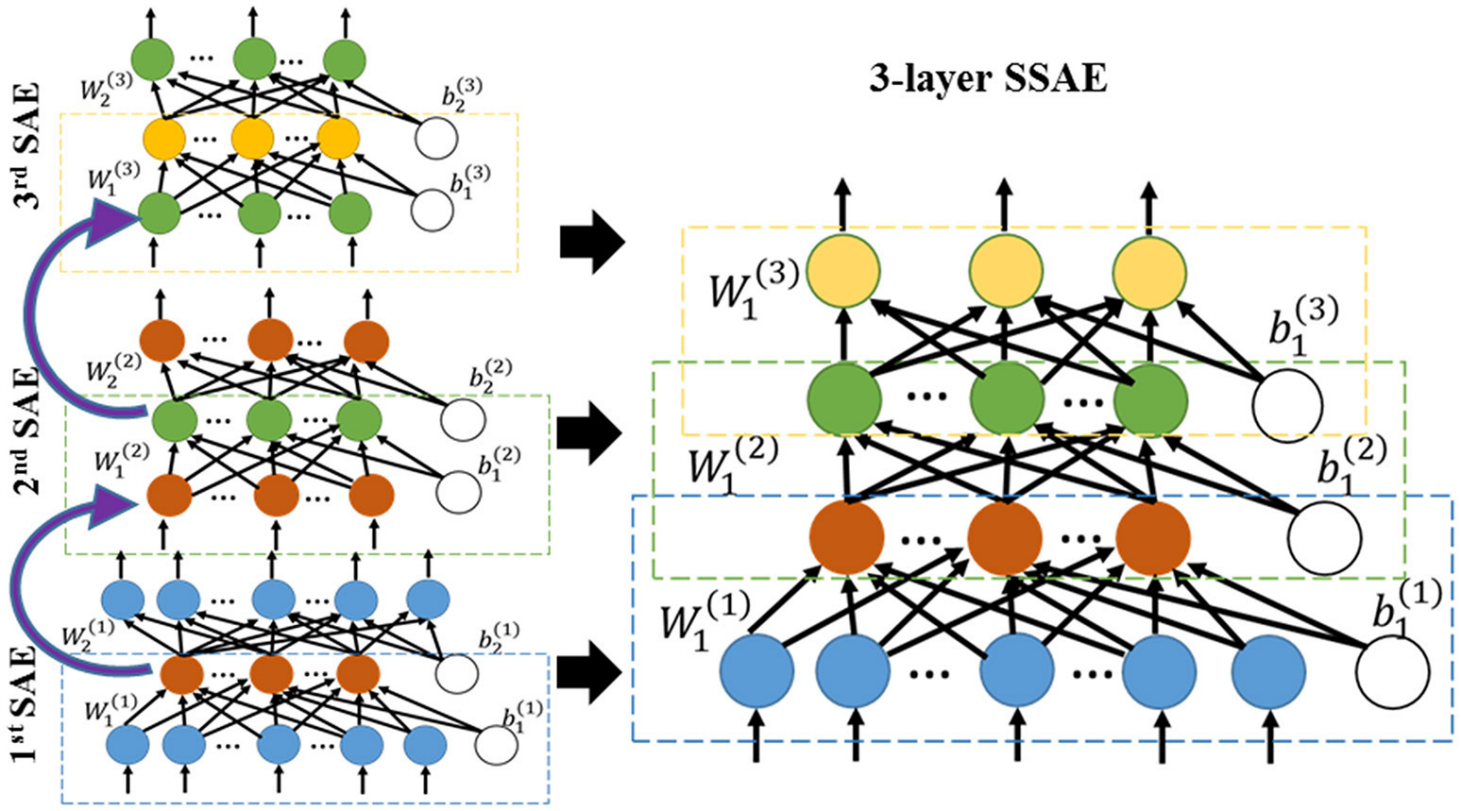
Three sparse AEs (encoding parts) are stacked together into a 3-layer SSAE.

In the first step of the training phase of our DTL-NN, we propose to learn healthy FC patterns through offline learning via SSAE. More specifically, we seek to train a SSAE prototype (Figure 1) using a large number of pre-collected healthy FC pattern data that are independent from the real classification task. In this way, these healthy FC patterns that are considered as prior knowledge would be represented by parameters (i.e., weights **W** and bias **b**) of the SSAE prototype. A SSAE model (Figure 2) is usually constructed by stacking multiple autoencoders (AE), which consists of one input layer, one hidden layer and one output layer. AE replicates its input at its output. Nodes between different layers of an AE are fully-connected. Here, we first describe the training process of individual AEs, then illustrate the stacking procedure of AEs into a SSAE model.

Assume an *n*-dimension FC features from a healthy subject ${\text{x}}^{h}=\left[x_{1}^{h},x_{2}^{h},\ldots ,x_{n}^{h}\right]$ where “*h*” denotes the healthy FC patterns. Also, assume the activation vector of κ hidden nodes ${\text{z}}^{h}=\left[z_{1}^{h},z_{2}^{h},\ldots ,z_{k}^{h}\right]$. The hidden nodes of an AE are activated using encoding weights **W**_**1**_ and bias **b**_**1**_ by ${\text{z}}^{h}=f\left({\text{W}}_{1}{\text{x}}^{h}+{\text{b}}_{1}\right)$. The output ${\hat{\text{x}}}^{h}=\left[{\hat{x}}_{1}^{h},{\hat{x}}_{2}^{h},\ldots ,{\hat{x}}_{n}^{h}\right]$ of an AE (i.e., reconstructed input vector) is presented by ${\hat{\text{x}}}^{h}=f\left({\text{W}}_{2}{\text{z}}^{h}+{\text{b}}_{2}\right)$ using the decoding weights **W**_**2**_ and bias **b**_**2**_. We further adopted an *L*_2_ regularized sparse AE in this work. The cost function of a sparse AE can be modeled by:

(1) $$\begin{array}{l} E\left(\text{W},\text{b}\right)=\frac{1}{p}\sum_{j=1}^{p}\sum_{i=1}^{n}{\left(\;{\hat{x}}_{ij}^{h}-x_{ij}^{h}\right)}^{2}+\lambda \\ \times \Omega_{\text{weights}}\;+\beta \times \Omega_{\text{sparsity}} \end{array}$$

In Equation (1), the first part is the mean squared error of AE's *i*^th^ output feature ${\hat{x}}_{ij}^{h}$ and *i*^th^ input feature $x_{ij}^{h}$ of sample *j*, and *p* is the sample size of the healthy FC patterns dataset. The second part of the cost function equation is the *L*_2_ regularization term $\Omega_{\text{weights}}=\frac{1}{2}\overset{n}{\underset{j=1}{\sum }}\overset{k}{\underset{i=1}{\sum }}{\left(\;w_{ij}\right)}^{2}$ on encoding weights *w*_*ij*_ from node *i* to node *j*, and λ is the *L*_2_ regularization penalty coefficient. The third part of the equation is the sparsity regularization term, where β is the coefficient for the sparsity regularization term and Ω_sparsity_ is the Kullback-Leibler (KL) divergence (Shin et al., 2013), defined by

(2) $$\Omega_{\text{sparsity}}=\sum_{i=1}^{k}\rho \log\frac{\rho }{{\rho_{i}}^{\prime }}+(1-\rho )log\frac{1-\rho }{1-{\rho_{i}}^{\prime }}$$

where ${\rho_{i}}^{\prime }=\frac{1}{p}\sum_{i=1}^{p}z_{i}(x_{j})$ is the average activation of the hidden node *i* over the training set. Sparsity parameter ρ is a pre-defined small fraction constant. Weights and bias of a *L*_2_ regularized sparse AE are initialized using a uniformly distributed random number between [−1, 1]. Weights and bias are optimized using a scaled conjugate gradient descent algorithm (Møller, 1993).

After training of individual AEs, we can stack multiple sparse AEs into an L layers SSAE model. In particular, the hidden layer of *l*^*th*^ sparse AE is connected to the input layer of (*l*+1)^th^ sparse AE, as shown in left panel of Figure 2. The encoding parts of three sparse AEs are stacked to form a SSAE. After the offline learning, we represented the healthy FC pattern knowledge by weights and bias of the SSAE prototype.

### Prior knowledge aided training

In the second step of the training phase, we incorporated the offline-learned prior knowledge (i.e., weights and bias) for a new subject FC classification task. The offline trained SSAE prototype was transferred to the new task—ASD classification in this work—and readied for further training. Conventionally, a DNN model requires a pre-training method (Hinton and Salakhutdinov, 2006; Bengio and LeCun, 2007) using the data from a classification task to initialize weights and bias of an SSAE. In contrast, our DTL-NN model utilized prior knowledge embedded in the offline learned SSAE prototype for the initialization instead of the classic pre-training approach. We assumed an independent dataset from *m* labeled subjects are represented as (**x**_1_, *y*_1_), (**x**_2_, *y*_2_), …, (**x**_*m*_, *y*_*m*_). In this work, this dataset is referred to FC patterns from ASD and healthy subjects, which are independent from the data in offline learning. Feature extraction was first performed using the SSAE prototype. The high-level features **z**_*i*_ were extracted by the L-layer SSAE prototype from the input **x**_*i*_. Then, the extracted high level features and the labels were used to train a softmax regression model. Given a high-level feature **z**_*i*_, the softmax regression model estimates the probabilities *p*(*y* = *j*|**z**_*i*_), *j* ∈ [1, …, Π] for a Π -class problem. The hypothesis of softmax regression is computed as follows:

(3) $$\begin{array}{l} h\left(z_{i}\right)=\frac{1}{\sum_{j=1}^{\Pi }e^{\theta_{j}^{T}{\text{z}}_{\text{i}}}}\left[\begin{array}{c} e^{\theta_{1}^{T}{\text{z}}_{\text{i}}}\\ e^{\theta_{2}^{T}{\text{z}}_{\text{i}}} \end{array}\right] \end{array}$$

The output of the hypothesis is a vector that contains Π probabilities, measuring the probability of the input samples for each class label. In the current work, we have only two categories (i.e., Π = 2). In Equation (3), **θ** is the coefficient vector of the model, which can be optimized by minimizing the cross entropy cost function:

(4) $$E\left(\theta \right)=-\frac{1}{m}\sum_{i=1}^{m}\sum_{j=1}^{\Pi }\left[h{\left(z_{i}\right)}_{j}\ln y_{ij}+(1-h{\left(z_{i}\right)}_{j})ln(1-y_{ij})\right],$$

where *y*_*ij*_ is the output of node *j* for sample *i* from the softmax regression model.

After the training of softmax regression model, we stacked this softmax regression model into the SSAE prototype from offline learning to form a DTL-NN model. Next, the whole DTL-NN model was optimized using supervised fine tuning (Hinton and Salakhutdinov, 2006). During this process, weights, bias from all layers of the SSAE and coefficients of the softmax regression were tuned simultaneously in each iteration using the scaled conjugate gradient descent scheme (Møller, 1993). Fine-tuning was terminated if the cost function goal was achieved or a maximum number of epochs occurred. Algorithm 1 in Supplementary Material summarizes the training algorithm of our DTL-NN.

### Rs-fMRI data from ABIDE repository

To test our hypothesis with the DTL-NN model, we chose the publicly available ABIDE repository (Craddock et al., 2013), which contains pre-processed rs-fMRI data from ASD and healthy subjects across all independent data sites. The rs-fMRI data were processed using Connectome Computation System (Xu et al., 2015) pipeline, which includes slice timing correction, motion realignment, and intensity normalization. Nuisance variable regression (Fox et al., 2005; Lund et al., 2005) was implemented through band-pass filtering and global signal regression strategies to clean confounding variations introduced by heartbeats and respirations, head motion, and low frequency scanner drifts. Furthermore, boundary-based rigid body and FMRIB's linear & nonlinear image registration tools (Andersson et al., 2008; Ratziu et al., 2008) were used to register functional to anatomical images. Then, both functional and anatomical images were normalized to a standard template space, the Automated Anatomical Labeling atlas (Tzourio-Mazoyer et al., 2002). The weights of FC was defined using Pearson's correlation between BOLD time series from two regions of interests (ROIs). This resulted in a 90 × 90 FC adjacency matrix, symmetric along diagonal, in which each entry represents the brain connectivity between each pair of ROIs. Site bias was corrected among different sites similar to an approach described previously by an ABIDE study (Heinsfeld et al., 2018). Specifically, a multiple regression analysis was conducted to control for potentially confounding variables (site, age, gender, and handedness). These variables were regressed from FC weights across connections among ROIs. When adjusting for the confounding factors, our regression model is:

$$\begin{array}{l} y=\beta_{0}+\beta_{1}x_{1}+\beta_{2}x_{2}+\beta_{3}x_{3}+\beta_{4}x_{4}+\varepsilon \end{array}$$

where *y* is the vector of FC weights, *x*_1_ to *x*_4_ represent the four explanatory variables, site, age, gender, and handedness. β_*i*_ is the intercept and the corresponding regression coefficients. ε is the residual vector. Using ordinary least squares (OLS) estimation, β_*i*_ was estimated. Then, these cofounding variables were regressed out from the FC weights. Next, the value of each FC was adjusted by using the difference between one site's mean value and mean values across all sites for the same FC. Example cases of FC maps before and after bias correction are presented in Supplemental Figure 1. For each individual subject, this resulted in 4,005 FC links that were considered as features.

### Evaluation of the classification model

**Figure 3:**
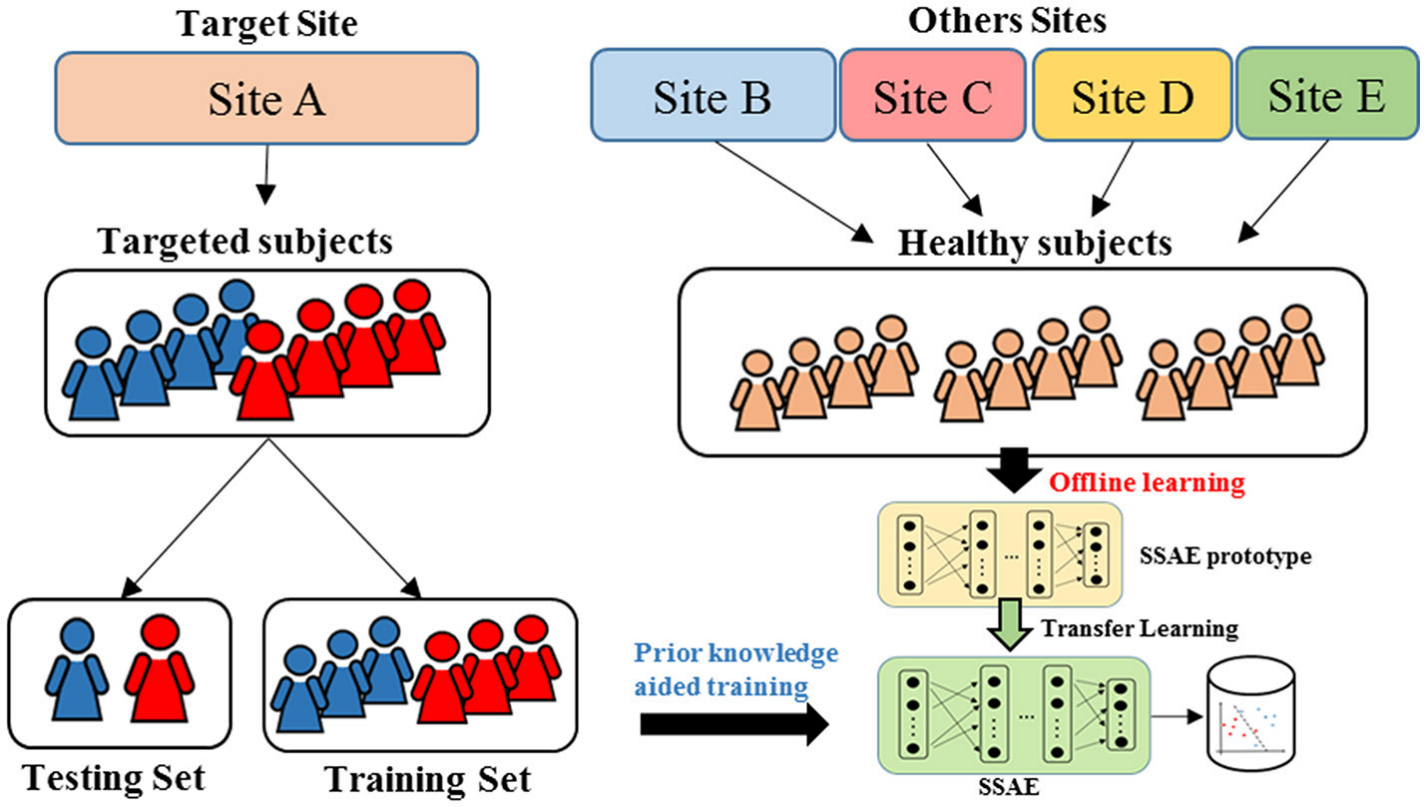
Cross validation scheme for DTL-NN model evaluation.

To design a reproducible study, we performed ASD classification experiments on four individual data sites with at least 60 subjects, including University of Michigan (UM), University of California, Los Angeles (UCLA), University of Utah School of Medicine (USM), and Katholieke Universiteit Leuven (LEUVEN). As shown in the Figure 3, when the models were evaluated on one data site, the independent healthy FC data from the remaining 16 sites from the ABIDE repository were accumulated as pre-collected data to train an SSAE prototype during offline learning. Then, we randomly split the data from the target site into training and testing set. The data from the training set were applied on an SSAE prototype in the prior knowledge aided training step. The hold-out testing data were then used to evaluate the trained DTL-NN model.

To measure the performance of ASD classification, we applied a k-fold cross-validation (CV) scheme. The whole target cohort was randomly divided into k equal sized portions. Of the k portions, one portion of data were held out for the model testing, and the remaining (k-1) portions were used for model training. This process was repeated k iterations until each of the k portions was evaluated once as the testing data. We evaluated the model based on the concatenated test labels and ground truth labels across k iterations. The training and testing data were separated proportionally according to the sample size of ASD and NC subjects in a stratified way. The performance of the classification was assessed using four diagnostic metrics: accuracy, sensitivity, specificity and area under receiver operating characteristic curve (AUC). Accuracy is measured by the percentage of correctly classified subjects within all subjects. Sensitivity is defined as the percentage of correctly classified ASD subjects within all ASD subjects, while specificity is represented by the percentage of correctly classified healthy subjects within all healthy subjects. Sensitivity is the ability of the classifier to correctly identify those ASD subjects (true positive rate), whereas specificity is the ability of the classifier to correctly identify healthy subjects (true negative rate). AUC reflects the diagnostic ability of a binary classifier system when its discrimination cutoff varies.

To investigate whether the classification performance was significantly increased by DTL-NN. A paired-sample *T*-test was conducted for our four performance metrics from the seven individual sites comparing the DTL-NN and DNN models.

### Optimization of ASD classification model

To provide a fair comparison between the performance of our DTL-NN model with conventional DNN (Bengio and LeCun, 2007), we first optimized the DNN model for each data site and then utilized the same configuration for both models. Thus, any difference in performance can be attributed to transfer learning. In this work, we adopted a 3-layer SSAE architecture from a previous ASD classification study (Guo et al., 2017). The SSAE model contained one input layer and three hidden layers. The number of nodes on the input layer was defined by the input features (i.e., 4,005 FC patterns). Empirical values (30, 50, 70, and 100) were tested to optimize the performance. A threshold of 10^−5^ was set for the cost function in (Equation 1). Different number of epochs (400, 500, 600, 700, and 800) were tested as a maximal training epoch (Kim et al., 2016), and the smallest number was chosen that achieved the best AUC performance. Regarding three sparsity hyperparameters of the model, sparsity regularization term β was selected from (1, 2, 3, and 4), while L_2_ regularized term coefficient λ was set to be 0.001, and Sparsity parameter ρ was selected from empirical values (0.001, 0.01, 0.05, and 0.1) based on AUC performance. Learning rate of back propagation algorithm for fine tuning is 0.01. The configurations used for DTL-NN and DNN are listed in Table 1.

**Table 1:** Configurations of DTL-NN and DNN.

**Site**	**Architecture**	**Regularization β**	**Sparsity ρ**	**Epochs**
UM	4005-100-100-100-2	1	0.1	800
UCLA	4005-70-70-70-2	2	0.01	400
USM	4005-30-30-30-2	2	0.01	400
LEUVEN	4005-50-50-50-2	3	0.01	400

### Identification of discriminative functional connections

To unveil FCs that were the most discriminative of ASD, we applied a feature ranking approach (Simonyan et al., 2013) designed for deep learning algorithms on our DTL-NN. Here, we calculated the partial derivatives $\frac{\partial y}{\partial FC_{ij}},\;i\neq j,i,j\in \left[1,2,\ldots ,90\right]$ of the labels with respect to the individual FC links from the brain connectome. The partial derivative for FC links can be represented by [**W**^(1)^ × **W**^(2)^ × …**W**^(*L*)^], where **W**^(*)^ is the optimized weights of individual layers of SSAE. A higher absolute value of the partial derivative indicates a higher level of the importance for ASD classification. The FC links were ranked inside every fold of k-fold CV, then the ranking weights of each FC link were accumulated across all folds of CV.

## Results

### Classification of ASD from individual data sites

We compared the performance of our DTL-NN model with DNN (Bengio and LeCun, 2007) and SVM learning approach across four data sites. The demographic information of the subjects is shown in Table 2.

**Table 2:** Demographic data of the healthy and autism spectrum disorder (ASD) subjects across seven data sites ordered by decreasing sample size.

**Site**	**Class**	**Target task sample size**	**Age (years)**	**Gender (male%)**	Handedness (right%)^*^	Mean full-scale IQ^*^	**Offline learning sample size**
UM	ASD	48	13.8 ± 2	81.30	75	107.6 ± 17.3	411
	Healthy	65	15 ± 3.7	75.40	83.10	109 ± 9.5	
UCLA	ASD	36	13.3 ± 3	94.40	88.90	102.4 ± 12.8	437
	Healthy	39	13.2 ± 1.8	84.60	92.30	106.4 ± 10.4	
USM	ASD	38	24.6 ± 9	100	92.10	99.7 ± 17.3	453
	Healthy	23	22.3 ± 7.9	100	95.70	115.5 ± 15.6	
LEUVEN	ASD	27	18 ± 5	92.60	88.90	109.4 ± 13.1	442
	Healthy	34	18.2 ± 5.1	85.30	85.30	114.8 ± 12.9	

*All ± values are mean ± SD*.

* *Results were calculated after removing missing data. Number of IQ missing value: UM, ASD-1, Healthy-3; UCLA, ASD-0, Healthy-0; USM, ASD-0, Healthy-0; LEUVEN, ASD-13, Healthy-19; Number of handedness missing value: UM, ASD-4, Healthy-3; UCLA, ASD-0, Healthy-0; USM, ASD-0, Healthy-0; LEUVEN, ASD-0, Healthy-0*.

The performance of ASD classification based 5-fold CV are illustrated in Table 3. We listed accuracy, sensitivity, specificity and AUC for four data sites that are ordered by their sample sizes. Across four individual data sites, SVM models achieved an average of 58.4% for accuracy, 59.8% for sensitivity, 57.5% for specificity and 0.63 for AUC. The poor performance might be due to the high dimensionality of FC maps in conjunction with the limited subjects. Similarly, DNN models classified ASD subjects with an average of 61.6%, 61.0%, 61.5% and 0.64 for accuracy, sensitivity, specificity and AUC, respectively. Compared to DNN and SVM, the DTL-NN model reached an average of 67.1% on accuracy, 65.7% on sensitivity, 68.3% on specificity and 0.71 on AUC, respectively. Our model significantly improved the ASD classification over the DNN model for accuracy (*p* = 0.03) and sensitivity (*p* = 0.04). For the UM site, that had the largest sample size (*N* = 113), DTL-NN achieved an accuracy of 67.2%, a 4.9% increase over the DNN model. Compared to DNN, our DTL-NN approach also produced better sensitivity, specificity, and AUC of 4.7, 6.3, and 0.05, respectively. The lowest performance improvement was noted from the UCLA site, where DTL-NN improved accuracy by only 1.6%, sensitivity by 0.7%, specificity by 3.4% and AUC by 0.05. The highest ASD classification performance metrics were observed from the USM site−70.4% accuracy, 72.5% sensitivity, 67.0% specificity and 0.73 AUC.

**Table 3:** ASD classification of four cohorts using different models.

**Site**	**Model**	**Accuracy (%)**	**Sensitivity (%)**	**Specificity (%)**	**AUC**
UM	SVM	60.5	63.8	58.2	0.60
	DNN	62.3	64.2	62.3	0.63
	DTL-NN	67.2	68.9	67.6	0.67
UCLA	SVM	53.9	51.7	55.9	0.56
	DNN	60.7	55.2	64.6	0.64
	DTL-NN	62.3	55.9	68.0	0.69
USM	SVM	63.6	66.8	61.3	0.67
	DNN	63.6	66.2	52.6	0.66
	DTL-NN	70.4	72.5	67.0	0.73
LEUVEN	SVM	55.7	57.0	54.7	0.59
	DNN	60.0	58.5	66.5	0.662
	DTL-NN	68.3	65.4	70.6	0.74

### Consistency test of DTL-NN with varying percentages of training data

**Figure 4:**
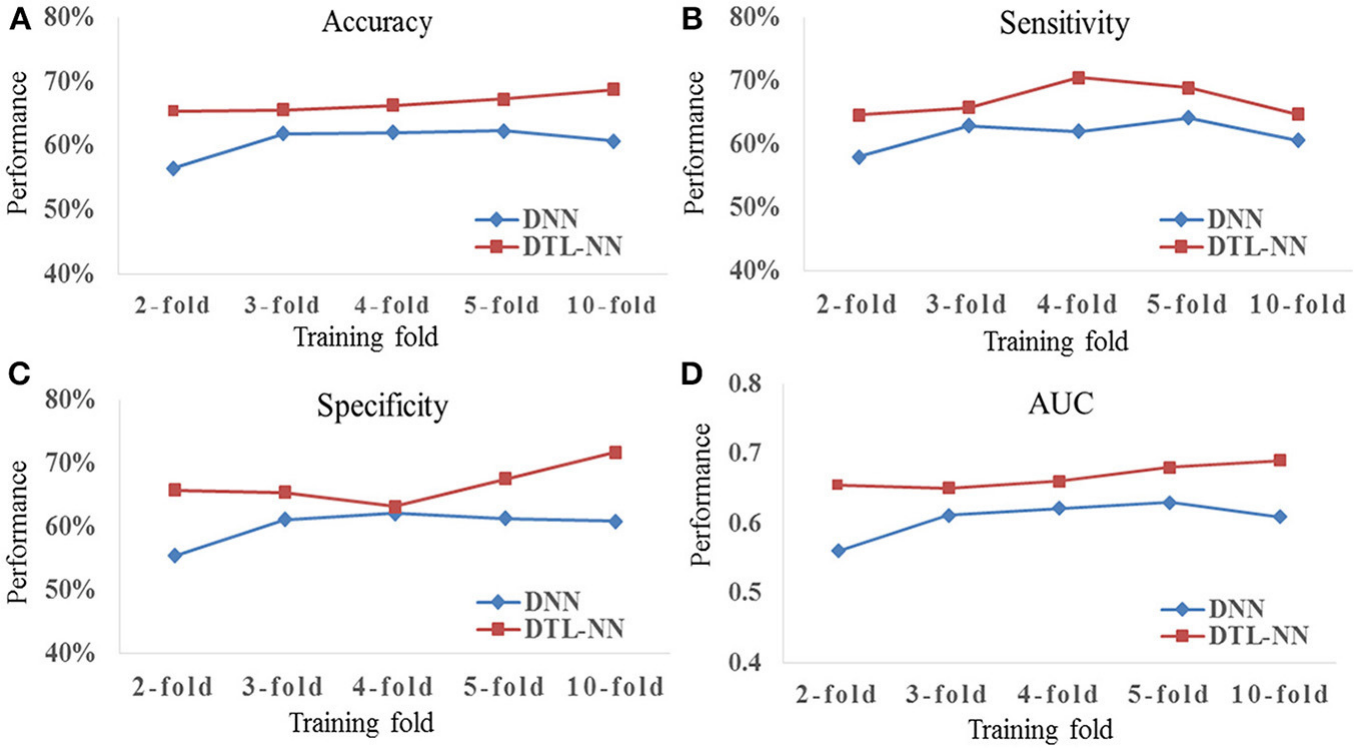
Classification performance **(A)** Accuracy, **(B)** Sensitivity, **(C)** Specificity, and **(D)** AUC of DNN and DTL-NN models with various percentages of training data.

We tested whether our proposed DTL-NN model could consistently improve classification performance with different subsampling cross-validation schemes. For these analyses, we only focused on the UM site that contained the largest sample size among our selected sites to ensure site sample size did not influence the results. Five commonly-used random percentages of training data (2-, 3-, 4-, 5-, and 10-fold) were tested. Figure 4 shows the mean accuracy, sensitivity, specificity and AUC of DNN and DTL-NN models using these five validation schemes. Overall, regardless of training percentage, DTL-NN showed consistent performance improvements over DNN.

### Discriminative FC patterns

**Figure 5:**
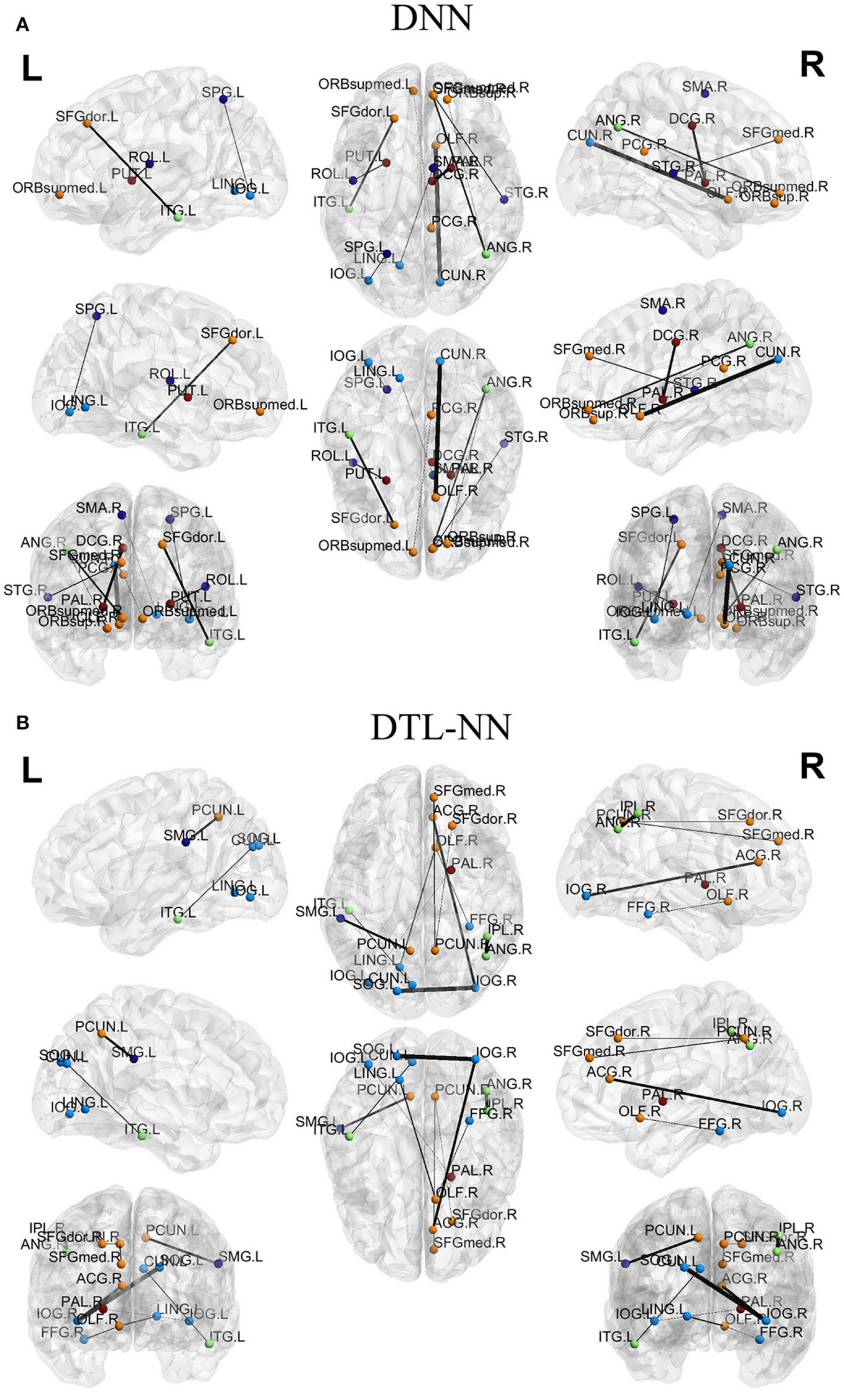
Top discriminative FCs identified by **(A)** DNN and **(B)** DTL-NN. The width of each segment/FC indicates the predictive strength (i.e., more predictive regions are wider).

We further explored the most discriminative FC patterns in ASD classification from the UM site. Table 4 summarizes the relevant FC features and abbreviations. Additional FC feature ranking results at three other data sites are now provided in the Supplemental Materials. In Figure 5, out of 4,005 FCs, we highlight the top 10 most discriminative ones identified by our DTL-NN and DNN approaches using BrainNet Viewer (Xia et al., 2013). We found that the FC between the left superior occipital gyrus and right inferior occipital gyrus was ranked as the top feature by the DTL-NN method based on data from UM site. This link was also ranked within top 10 discriminative FC links at UCLA, USM and LEUVEN sites by using DTL-NN model (Supplemental Tables 1–3). These nodes have been reported by multiple previous ASD studies (Just et al., 2006; Ha et al., 2015; Heinsfeld et al., 2018). In contrast, this same link between the left superior occipital gyrus and right inferior occipital gyrus was only selected as a high discriminative feature by traditional DNN from one (UCLA) site (Supplemental Table 1). The top feature revealed by DNN was the link between right olfactory and right cuneus regions. These regions were also associated with ASD previously (May et al., 2011), indicating the strong data mining ability of DNN. Of note, the fusiform gyrus area discovered by our DTL-NN model was also highlighted by a recent study (Heinsfeld et al., 2018), but was not ranked high by the DNN model.

**Table 4:** Top 10 discriminative FC features for DNN and DTL-NN models.

**Top 10 discriminative FC features of UM site**			
**Brain Region A**	**Abbr**.	**Brain Region B**	**Abrr**.
**DNN**			
Olfactory right	OLF-R	Cuneus right	CUN-R
Superior frontal gyrus (dorsal) left	SFGdor-L	Inferior temporal gyrus left	ITG-L
Middle cingulate gyrus right	MCG-R	Pallidum right	PAL-R
Orbitofrontal cortex (medial) right	ORBmed-R	Angular gyrus right	ANG-R
Rolandic operculum left	ROL-L	Putamen left	PUT-L
Superior frontal gyrus (medial) right	SFGmed-R	Superior temporal gyrus right	STG-R
Supplementary motor area right	SMA-R	Lingual gyrus left	LING-L
Inferior occipital gyrus left	IOG-L	Superior parietal gyrus left	SPG-L
Orbitofrontal cortex (superior) right	ORBsup-R	Angular gyrus right	ANG-R
Orbitofrontal cortex (medial) left	ORBmed-L	Posterior cingulate gyrus right	PCG-R
**DTL-NN**			
Superior occipital gyrus left	SOG-L	Inferior occipital gyrus right	IOG-R
Inferior parietal lobule right	IPL-R	Angular gyrus right	ANG-R
Supramarginal gyrus left	SMG-L	Precuneus left	PCUN-L
Anterior cingulate gyrus right	ACG-R	Inferior occipital gyrus right	IOG-R
Olfactory right	OLF-R	Lingual gyrus left	LING-L
Cuneus left	CUN-L	Inferior temporal gyrus left	ITG-L
Superior frontal gyrus (medial) right	SFGmed-R	Precuneus right	PCUN-R
Olfactory right	OLF-R	Fusiform gyrus right	FFG-R
Superior frontal gyrus (dorsal) right	SFGdor-R	Precuneus right	PCUN-R
Inferior occipital gyrus left	IOG-L	Pallidum right	PAL-R

## Discussion

We demonstrated that healthy FC patterns can be learned by neural networks from an existing large database and this knowledge can be transferred to enhance an ASD classification task that also replies on FC patterns. Inspired by successful use of transfer learning strategy in other fields, we developed a DTL-NN model that effectively utilizes healthy FC patterns from an existing database. Unlike prior DNN studies, our DTL-NN model is not only trained with the FC samples from a given target task, but additionally trained using pre-collected healthy FC patterns. Utilizing the same model configurations, our proposed DTL-NN model was able to achieve significantly improved classification performance on varying independent tasks.

To utilize deep learning techniques effectively, we must first comprehend their limitations. Before recent advances in deep learning, multi-layer neural network models were often characterized by a “local minima problem,” which negatively impacted the performance of the model. A pre-training strategy (Hinton and Salakhutdinov, 2006) proposed in 2006 mitigated this “local minima problem.” This was done by initializing the neural network model to a point in the parameter space that is appropriate for further supervised training in order to achieve a lower minimum of the cost function (Bengio and LeCun, 2007). But, this pre-training strategy relied on sufficient training data. As discussed earlier, large scale data collection in brain rs-fMRI studies is challenging. Thus, transfer learning (Pan and Yang, 2010) that is designed for tasks with limited data, merits our attention.

Here, we proposed the DTL-NN model and evaluated it using the ABIDE data repository. As shown in Table 3, the DTL-NN model performed well, aided by prior knowledge, on the ASD classification task using FC patterns. The reproducible results across multiple data sites supported our hypothesis that a large scale of healthy FC patterns data could be transferred to enhance brain FC patterns classification from an independent study. Our best ASD classification performance was achieved by DTL-NN from the USM site with 70.4% accuracy, 72.5% sensitivity, 67.0% specificity and 0.7 AUC. This is comparable to a recent ASD study (Heinsfeld et al., 2018) using a deep learning algorithm. The difference is that our DTL-NN model was able to achieve similar performance by using only a modest size of samples, while previous work required a large number of data for model training.

We tested the consistency of DTL-NN model by varying the data splitting strategy on the largest UM site. The consistent improvement achieved by DTL-NN over five training scenarios indicated the robust efficacy of transfer learning (Figure 4). Furthermore, we ranked the 10 most discriminative FC patterns identified by the DTL-NN and DNN models (Figure 5; Table 4). The FC between left superior occipital gyrus and right inferior occipital gyrus was consistently ranked as the top discriminative feature by the DTL-NN method across four study sites, while this FC link was only selected by DNN from one site (Table 4, Supplemental Tables 1–3). The consistency of feature selection by using DTL-NN is better than DNN across different sites. This might be due to the same offline learning of DTL-NN using healthy FC patterns. In addition, our DTL-NN also highlighted a FC feature connected to the fusiform gyrus, an area previously associated with ASD development (Heinsfeld et al., 2018), which was not emphasized by the DNN model from the UM and USM sites. This suggests that the DTL-NN approach discovers more meaningful brain features by utilizing prior knowledge learned from healthy subjects compared to the DNN model that solely relies on the targeted dataset.

We only utilized pre-collected data for offline learning from healthy subjects because such data were relatively easy to acquire. For future work, even larger datasets for offline learning are possible by integrating all healthy subjects from additional large-scale brain studies such as the Human Connectome Project, ADHD-200 and ADNI. As a preliminary study to demonstrate the capability of transfer learning in neuroimaging studies, we narrowed down our research scope and focused on only healthy FC patterns in offline learning. In fact, additional FC patterns from other disease groups could also be included in offline learning. We have made an initial attempt on apply both healthy and ASD subjects to aid the classification of other brain conditions. For example, we utilized FC patterns from both healthy and ASD subjects in our recent study (He et al., 2018) on early prediction of cognitive deficits in a cohort of very preterm infants. By adopting the transfer learning strategy, a total of 884 independent FC patterns from ABIDE were applied to train a SSAE model in an unsupervised manner. Then, we transferred the SSAE model to perform dimension reduction for the FC patterns of our preterm cohort. With the high-level features of FC patterns, our SVM model predicted cognitive deficits at 2 years corrected age for very premature infants with an accuracy of 70.6% and AUC of 0.76. However, we realize that several confounding factors may impact performance. For example, as more samples from disease groups are added into offline learning, whether the performance change is due to an increase in sample size or disease condition remains unclear. To answer such a question, a comprehensive analysis and discussion will be necessary. Thus, whether data from subjects with relevant or disparate conditions would enhance classification remains an interesting topic for future inquiry.

Our study has several limitations. First, when knowledge is based on one type of feature (e.g., FC patterns), only the same type of feature can be represented by the knowledge. The learned knowledge is unlikely to be transferable to other types of features. Second, transfer learning cannot replace the necessary data collection in neuroscience studies. As such, the DTL-NN model may not be able to improve the performance of a classification model that is trained with sufficient data. Thus, the real value of our approach lies in its application to smaller studies, especially rare neurological disorders, where performing large studies is very challenging. Third, we only considered FC patterns as a full-weighted graph in the current study. FC patterns could also be represented by other networks (e.g., dynamic FC networks, Hutchison et al., 2013 or hypothesis-driven subnet, He and Parikh, 2016). Whether our approach can be applied on other FC networks represents an interesting future inquiry. Last, due to the diverse complexity of classification problems, it is difficult to estimate how many healthy FC patterns are sufficient in transfer learning to achieve optimal performance. The sample sizes of healthy controls for four data sites in the current work were all approximately 400. It will be interesting to explore in future studies if a larger set of healthy FC patterns (e.g., 5,000) could further improve classification performance.

In summary, we developed a novel DTL-NN framework by utilizing healthy FC patterns to facilitate the application of deep learning models for smaller neuroimaging rs-fMRI studies and demonstrated enhanced ASD classification as compared to DNN models. The significantly improved performance was observed irrespective of site sample size and was reproducible among various subsampling schemes. Our results offer a proof of concept for the use of DTL-NN models over conventional DNN models to enhance diagnosis or prediction of rare diseases and other conditions where studying a large cohort remains challenging.

## Author contributions

HL and LH conceived and designed the experiments. HL and LH performed the experiments. HL, LH, and NP wrote the paper.

### Conflict of interest statement

The authors declare that the research was conducted in the absence of any commercial or financial relationships that could be construed as a potential conflict of interest.
