# Supplementary material for: A Novel Transfer Learning Approach to Enhance Deep Neural Network Classification of Brain Functional Connectomes
Source: Front Neurosci. 2018 Jul 24;12:491. doi: 10.3389/fnins.2018.00491 (PMC6066582; doi:10.3389/fnins.2018.00491)

Supplemental Table 1. Top 10 discriminative FC features for DNN and DTL-NN models of UCLA site.

**Top 10 Discriminative FC features of UCLA site**			
**DNN**			
**Brain Region A**	**Abbr.**	**Brain Region B**	**Abrr.**
Hippocampus right	HIP-R	Inferior occipital gyrus left	IOG-L
Posterior cingulate gyrus left	PCG-L	Hippocampus right	HIP-R
ParaHippocampal gyrus left	PHG-L	Inferior occipital gyrus left	IOG-L
Middle temporal gyrus right	MTG-R	Temporal pole (middle) right	TPOmid-R
Olfactory left	OLF-L	Precuneus right	PCUN-R
Posterior cingulate gyrus left	PCG-L	Supramarginal gyrus right	SMG-R
Superior occipital gyrus left	SOG-L	Inferior occipital gyrus right	IOG-R
Olfactory right	OLF-R	Inferior occipital gyrus right	IOG-R
Superior frontal gyrus (medial) left	SFGmed-L	Amygdala right	AMYG-R
Anterior cingulate gyrus left	ACG-L	Precuneus right	PCUN-R
**DTL-NN**			
**Brain Region A**	**Abbr.**	**Brain Region B**	**Abrr.**
Superior parietal gyrus left	SPG-L	Precuneus right	PCUN-R
Superior occipital gyrus left	SOG-L	Inferior occipital gyrus right	IOG-R
Superior frontal gyrus (dorsal) right	SFGdor-R	Superior occipital gyrus right	SOG-R
Amygdala left	AMYG-L	Superior temporal gyrus right	STG-R
Superior frontal gyrus (dorsal) right	SFGdor-R	Superior occipital gyrus left	SOG-L
Superior occipital gyrus left	SOG-L	Paracentral lobule left	PCL-L
Posterior cingulate gyrus left	PCG-L	Superior parietal gyrus left	SPG-L
Superior parietal gyrus left	SPG-L	Angular gyrus right	ANG-R
Middle cingulate gyrus right	MCG-R	Paracentral lobule right	PCL-R
Superior occipital gyrus left	SOG-L	Precuneus left	PCUN-L

Supplemental Table 2. Top 10 discriminative FC features for DNN and DTL-NN models of USM site.

Top 10 Discriminative FC features of USM site			
**DNN**			
**Brain Region A**	**Abbr.**	**Brain Region B**	**Abrr.**
Superior frontal gyrus (dorsal) right	SFGdor-R	Inferior frontal gyrus (opercular) right	IFGoperc-R
Middle frontal gyrus left	MFG-L	Thalamus left	THA-L
Orbitofrontal cortex (middle) right	ORBmid-R	Calcarine cortex right	CAL-R
Inferior frontal gyrus (triangular) right	IFGtriang-R	Rectus gyrus right	REC-R
Putamen left	PUT-L	Inferior temporal gyrus left	ITG-L
Inferior parietal lobule left	IPL-L	Putamen left	PUT-L
Orbitofrontal cortex (middle) right	ORBmid-R	Amygdala right	AMYG-R
Orbitofrontal cortex (middle) left	ORBmid-L	Posterior cingulate gyrus right	PCG-R
Paracentral lobule left	PCL-L	Thalamus right	THA-R
Rolandic operculum right	ROL-R	Postcentral gyrus right	PoCG-R
**DTL-NN**			
**Brain Region A**	**Abbr.**	**Brain Region B**	**Abrr.**
Putamen left	PUT-L	Inferior temporal gyrus left	ITG-L
Superior parietal gyrus left	SPG-L	Precuneus right	PCUN-R
Superior occipital gyrus left	SOG-L	Inferior occipital gyrus right	IOG-R
Superior frontal gyrus (dorsal) right	SFGdor-R	Superior occipital gyrus right	SOG-R
Supplementary motor area right	SMA-R	Middle occipital gyrus left	MOG-L
Paracentral lobule right	PCL-R	Thalamus left	THA-L
Superior frontal gyrus (medial) right	SFGmed-R	Middle cingulate gyrus left	MCG-L
Superior occipital gyrus right	SOG-R	Fusiform gyrus left	FFG-L
Precentral gyrus right	PreCG-R	Inferior parietal lobule left	IPL-L
Precentral gyrus right	PreCG-R	Angular gyrus left	ANG-L

Supplemental Table 3. Top 10 discriminative FC features for DNN and DTL-NN models of LEUVEN site.

**Top 10 Discriminative FC features of LEUVEN site**			
**DNN**			
**Brain Region A**	**Abbr.**	**Brain Region B**	**Abrr.**
Precentral gyrus left	PreCG-L	Superior parietal gyrus right	SPG-R
Supplementary motor area left	SMA-L	Paracentral lobule left	PCL-L
Postcentral gyrus left	PoCG-L	Superior parietal gyrus right	SPG-R
Postcentral gyrus right	PoCG-R	Paracentral lobule right	PCL-R
Posterior cingulate gyrus left	PCG-L	Caudate right	CAU-R
Precentral gyrus left	PreCG-L	Precentral gyrus right	PreCG-R
Anterior cingulate gyrus left	ACG-L	Paracentral lobule left	PCL-L
Superior frontal gyrus (dorsal) left	SFGdor-L	Paracentral lobule left	PCL-L
Anterior cingulate gyrus left	ACG-L	Paracentral lobule right	PCL-R
Anterior cingulate gyrus right	ACG-R	Postcentral gyrus left	PoCG-L
**DTL-NN**			
**Brain Region A**	**Abbr.**	**Brain Region B**	**Abrr.**
Anterior cingulate gyrus left	ACG-L	Paracentral lobule right	PCL-R
Supplementary motor area left	SMA-L	Paracentral lobule left	PCL-L
Superior occipital gyrus left	SOG-L	Inferior occipital gyrus right	IOG-R
Superior frontal gyrus (dorsal) left	SFGdor-L	Heschl gyrus right	HES-R
Orbitofrontal cortex (middle) left	ORBmid-L	Lingual gyrus right	LING-R
Orbitofrontal cortex (superior) left	ORBsup-L	Precuneus right	PCUN-R
Middle cingulate gyrus right	MCG-R	Paracentral lobule right	PCL-R
Anterior cingulate gyrus right	ACG-R	Paracentral lobule right	PCL-R
Anterior cingulate gyrus left	ACG-L	Paracentral lobule left	PCL-L
Inferior frontal gyrus (opercular) right	IFGoperc-R	Temporal pole (middle) right	TPOmid-R

Supplemental Figure 1. FC maps examples before and after site bias normalization at UM site

(**A**) Before site bias normalization

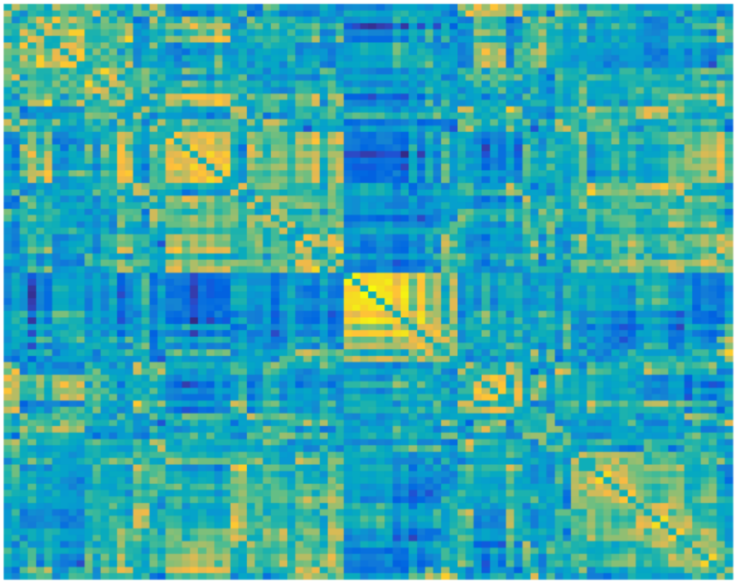

(**B**) After site bias normalization

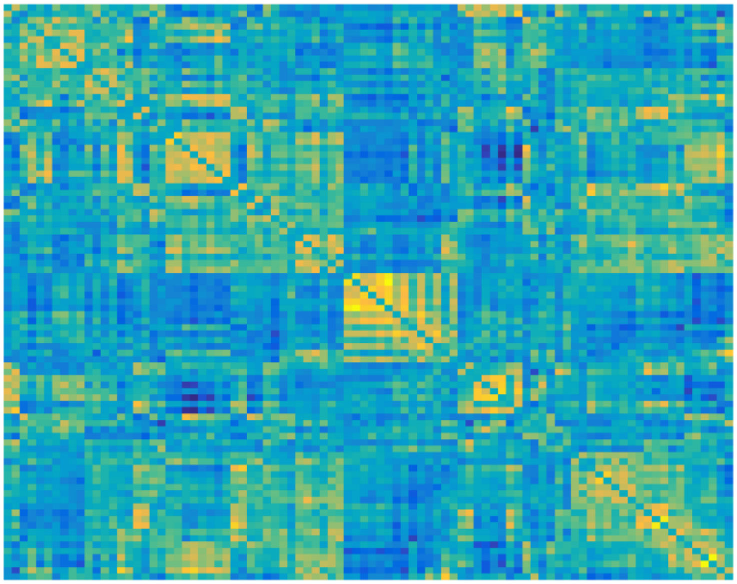

Supplement: Supplementary file 1 [file Data_Sheet_1.pdf]
