# Supplementary material for: A Novel Transfer Learning Approach to Enhance Deep Neural Network Classification of Brain Functional Connectomes
Source: Front Neurosci. 2018 Jul 24;12:491. doi: 10.3389/fnins.2018.00491 (PMC6066582; doi:10.3389/fnins.2018.00491)
Supplement: Supplementary file 2 [file Data_Sheet_2.docx]

**Algorithm 1**

| **Learning Algorithm: DTL-NN** |
| --- |
| **Input 1:** Healthy data$\{\mathbf{x}_{1}^{h},\mathbf{x}_{2}^{h},\ldots,\mathbf{x}_{p}^{h}\}$  **Input 2:** Task labeled data $\{\left( \mathbf{x}_{1},y_{1} \right), \left( \mathbf{x}_{2},y_{2} \right),\ldots, \left( \mathbf{x}_{m},y_{m} \right)\}$ |
| **Output:** DTL-NN model $\{\boldsymbol{W,b,\theta}\}$ |
| *# offline learning*  ***for k=1:L layers of SSAE*** |
| ***Minimize***$E\left( \mathbf{W},\mathbf{b} \right)=\frac{1}{p}\sum_{j=1}^{p} \sum_{i=1}^{n} {( \hat{x}_{ij}^{h}-x_{ij}^{h})}^{2}+\lambda\times\Omega_{\mathrm{weights}} +\beta\times\Omega_{\mathrm{sparsity}}$ |
| ***End*** |
| *# prior knowledge aided classification*  ***Calculate high-level features for targeted subjects*** $\mathbf{z}_{i}$  ***Supervised learning of softmax regression by optimizing***  $E\left( \boldsymbol{\theta} \right)\mathbf{=-}\frac{1}{m}\sum_{i=1}^{m} \sum_{j=1}^{\Pi} [{h\left( \boldsymbol{z}_{i} \right)}_{j}\ln y_{ij}+(1-{h(\boldsymbol{z}_{i})}_{j})ln(1-y_{ij})]$ |
| ***Fine tuning of the DTL-NN with task data***  ***Return*** $\{\boldsymbol{W,b,\theta}\}$ |
